# Overexpression of the nucleoporin Nup88 stimulates migration and invasion of HeLa cells

**DOI:** 10.1007/s00418-021-02020-w

**Published:** 2021-07-31

**Authors:** Masaki Makise, Ryota Uchimura, Kumiko Higashi, Yasumi Mashiki, Rikako Shiraishi, Yuumi Shutoku, Akihiko Kuniyasu

**Affiliations:** Faculty of Pharmaceutical Sciences, Sojo University, 4-22-1 Ikeda, Nishi-ku, Kumamoto, 860-0082 Japan

**Keywords:** Nup88, HeLa cells, Migration, Invasion, NF-κB, MMP-12

## Abstract

Elevated expression of the nucleoporin Nup88, a constituent of the nuclear pore complex, is seen in various types of malignant tumors, but whether this overexpression contributes to the malignant phenotype has yet to be determined. Here, we investigated the effect of the overexpression of Nup88 on the migration and invasion of cervical cancer HeLa cells. The overexpression of Nup88 promoted a slight but significant increase in both migration and invasion, whereas knockdown of Nup88 by RNA interference suppressed these phenotypes. The observed phenotypes in Nup88-overexpressing HeLa cells were not due to the progression of the epithelial-to-mesenchymal transition or activation of NF-κB, which are known to be important for cell migration and invasion. Instead, we identified an upregulation of matrix metalloproteinase-12 (MMP-12) at both the gene and protein levels in Nup88-overexpressing HeLa cells. Upregulation of MMP-12 protein by the overexpression of Nup88 was also observed in one other cervical cancer cell line and two prostate cancer cell lines but not 293 cells. Treatment with a selective inhibitor against MMP-12 enzymatic activity significantly suppressed the invasive ability of HeLa cells induced by Nup88 overexpression. Taken together, our results suggest that overexpression of Nup88 can stimulate malignant phenotypes including invasive ability, which is promoted by MMP-12 expression.

**Supplementary Information:**

The online version contains supplementary material available at 10.1007/s00418-021-02020-w.

## Introduction

Nuclear pore complexes (NPCs) are channels that penetrate the nuclear envelope to mediate the selective trafficking of macromolecules between the cytoplasm and the nucleus in eukaryotic cells. The vertebrate NPC is made up of about 30 distinct proteins termed nucleoporins (Nups) (Cronshaw et al. 2002). Approximately one-third of Nups comprise phenylalanine-glycine (FG)-rich repeats, which combine to form a meshwork in the center of the NPC. This meshwork serves as a physical barrier to limit the passive diffusion of macromolecules through the NPC. Moreover, nuclear transport receptors, or karyopherins, bind to NPC via these FG-repeats to modulate the selective transport of proteins and mRNAs. A variety of Nups have been well-characterized with respect to nuclear trafficking. Recent investigations, however, suggest that NPCs play additional roles in cellular processes, including cancer progression (Simon and Rout 2014; Snow and Paschal 2014).

Nup88 is a non-FG Nup primarily found on the cytoplasmic face of NPCs. While Nup88 is involved in nuclear export coordinated with Nup98 and Nup214, only Nup88 has been found to be highly expressed in a variety of cancer cell lines and tumors. The expression level of Nup88 has been reported to be associated with both tumorigenesis and malignant transformation (Xu and Powers 2009). Indeed, overexpression of Nup88 appears to regulate the activity of NF-κB, a ubiquitous transcriptional factor involved in cancer progression, at the level of nucleocytoplasmic transport in tumor cells (Takahashi et al. 2008). Elevated expression of Nup88 in a mouse model was shown to induce intestinal cancer accompanied by aneuploidy and chromosome instability (Naylor et al. 2016). Moreover, it was reported that Nup88 exhibits a high level of expression at the invasive margin and areas of vascular invasion in primary and metastatic colorectal cancers (Emterling et al. 2003). However, whether or how the upregulated expression of Nup88 contributes to cancer has yet to be fully established.

Degradation of the extracellular matrix (ECM) surrounding tumor tissues is critical for malignant transformation (Egeblad and Werb 2002; Fingleton 2006). Matrix metalloproteases (MMPs), a family of calcium-dependent zinc-containing proteases, are principally responsible for ECM degradation. In humans, more than 23 MMPs have been identified (Bonnans et al. 2014), and their expression is thought to be controlled by transcriptional regulation (Yan and Boyd 2007). Most MMPs are secreted from cells as inactive zymogens and then subsequently activated in the extracellular space by serine proteinases or other activated MMPs (Egeblad and Werb 2002). MMPs are upregulated in many types of human cancers, and their expression is closely associated with epithelial-to-mesenchymal transition (EMT) and poor prognosis. Intriguingly, however, in addition to their pro-tumorigenic effects, some MMPs can also elicit a beneficial and protective effect in suppressing tumor progression (Martin and Matrisian 2007). One such type of MMP is MMP-12, which was initially identified from alveolar macrophages (Shapiro et al. 1993). Elevated expression of MMP-12 was reported to be associated with cancer malignancy in non-small cell lung cancer, hepatocellular carcinoma, cutaneous melanoma, and cervical carcinoma (Hofmann et al. 2005; Ng et al. 2011; Vazquez-Ortiz et al. 2005; Zhang et al. 2015). By contrast, its overexpression in gastric and colorectal cancer was reported to be associated with increased survival and improved prognosis (Cheng et al. 2010; Yang et al. 2001). Thus, the effect of individual MMPs in cancer progression appears to be dependent on the type of tumor cells in which they are expressed.

In the present study, we aimed to gain insight into the role of Nup88 during tumor malignancy. Specifically, we examined the influence of the overexpression of Nup88 on both migration and invasion in HeLa cells. Our findings demonstrate that Nup88 contributes to the malignant phenotype via increased expression of MMP-12 in HeLa cells.

## Materials and methods

### Plasmids and antibodies

A plasmid designed to express green fluorescent protein (GFP)-tagged Nup88 under the control of a cytomegalovirus (CMV) promoter was generated by cloning Nup88 cDNA into pEGFP-N2 (Clontech, Mountain View, CA, USA). A construct with DDK-tagged Twist1 (#RC202920) was purchased from OriGene (Rockville, MD, USA). Rabbit polyclonal anti-GFP (#598) and anti-α-Tubulin (#PM054) were purchased from Medical & Biological Laboratories (Tokyo, Japan). Rabbit polyclonal anti-Snail1 (#sc-28199), rabbit polyclonal anti-Twist1 (#sc-15393), and mouse monoclonal anti-vimentin (#sc-6260) were purchased from Santa Cruz Biotechnology (Dallas, TX, USA). Mouse monoclonal anti-CD324 (E-cadherin) (#562869) was obtained from BD Biosciences (Franklin Lakes, NJ, USA). Mouse monoclonal Lamin A/C (#4777S) and rabbit polyclonal anti-RELA (p65 subunit of NF-κB, #10745-1-AP) were purchased from Cell Signaling Technology (Danvers, MA,, USA) and Proteintech (Tokyo, Japan), respectively. Rabbit monoclonal anti-MMP-12 was purchased from Abcam (#ab52897; Cambridge, UK). Peroxidase-conjugated goat anti-rabbit IgG (#111-036-144) and anti-mouse IgG (#115-036-146) were purchased from Jackson ImmunoResearch Laboratories, Inc. (West Grove, PA, USA).

### Cell culture and cell lines

Cells were routinely cultured in Dulbecco’s modified Eagle’s medium (DMEM) or Roswell Park Memorial Institute (RPMI)-1640 supplemented with 10% fetal bovine serum (FBS) and penicillin/streptomycin in a humidified atmosphere containing 5% CO_2_ at 37 °C. Cells were temporarily cultured in a medium with decreased FBS (f.c. 1%) for recovery of secreted MMP-12 in the medium. T-REx HeLa cells containing a single Flp recombination site in the genome were established from HeLa R19 cells (Kaiser et al. 2008). MCF-7 (#ATCC^®^ HTB-22™) and T-REx 293 cells (#R71007) were purchased from American Type Culture Collection (Manassas, VA, USA) and Thermo Fisher Scientific (Waltham, MA, USA), respectively. CaSki (#RCB1947), LNCap (#RCB2144), and PC-3 (#RCB2145) cells were purchased from RIKEN BRC (Tsukuba, Japan). Stable T-REx HeLa cells that express GFP and GFP-tagged Nup88 under the control of the Tet-on promoter were established previously (Makise et al. 2018). The T-REx 293 stable cell lines were established according to a previous report (Makise et al. 2018). Protein expression in T-REx HeLa and 293 stable cell lines were induced using a medium containing 1 µg/ml doxycycline (DOX) for 24–48 h. To establish MCF-7 and CaSki stable cell lines that express GFP or GFP-fused Nup88, cells transfected with the corresponding expression plasmid were cultured continuously in a medium containing 200–800 µg/ml G418 for about 1 month. Any colonies that formed were isolated, and heterologous protein expression was examined by immunoblotting.

### Transfection of plasmids and siRNAs

Plasmids and siRNAs were transfected using Lipofectamine LTX and Lipofectamine RNAiMax reagent, respectively (Life Technologies, Carlsbad, CA, USA), according to the manufacturer’s instructions. siRNAs for control (#4390843), NUP88 (ID: s9779), or RELA (p65) (ID: s11915) were purchased from Ambion (Foster City, CA, USA).

### Immunoblotting

Whole-cell lysates were prepared with radioimmunoprecipitation assay (RIPA) buffer (50 mM Tris/HCl pH 8.0, 150 mM NaCl, 5 mM EDTA, 1% Nonidet P-40, 0.5% sodium deoxycholate, 0.1% sodium dodecyl sulphate [SDS]). Soluble proteins in the culture medium were precipitated by the addition of 10% trichloroacetic acid and then dissolved in RIPA buffer. Nuclear or cytoplasmic fractions were prepared using a Nuclear/Cytosol Fractionation Kit (BioVision, Milpitas, CA, USA) according to the manufacturer’s instructions. The resultant fractions were subjected to SDS–polyacrylamide gel electrophoresis (SDS-PAGE). Proteins were generally electrophoresed through 10% polyacrylamide gels. However, Nup214, E-cadherin, and Twist1 and Snail1 were analyzed using 6%, 8%, and 12% polyacrylamide gels, respectively. Proteins separated in the gel were electroblotted onto an Immobilon-P transfer membrane (Merck Millipore, Burlington, MA, USA). The membrane was blocked with blocking buffer (5% skim milk, 0.05% Tween 20 in phosphate-buffered saline [PBS]) for 30 min at room temperature and then incubated with primary antibodies. All primary antibodies used were diluted 1:1000 with PBS containing 1% skim milk and 0.05% Tween 20 and then incubated with the membrane at 4 °C overnight with gentle mixing. After incubation, the membrane was washed three times with wash buffer (0.05% Tween 20 in PBS) at 5 min intervals and then incubated with peroxidase-conjugated secondary antibodies at 4 °C for 1 h. Chemiluminescence was generated by the addition of Luminata Crescendo Western HRP (horseradish peroxidase) substrate (Merck Millipore) to the membrane, and cross-reacting bands were detected using a LAS2000 imaging analyzer (Fujifilm, Tokyo, Japan).

### Scratch wound healing assay

Scratch wound healing assays were performed as described previously (Liang et al. 2007)*.* Briefly, cells forming a confluent layer in a well of the collagen-coated 24-well culture plate (AGC Techno Glass, Tokyo, Japan) were scraped with a P200 pipette tip in a straight line to generate a gap of 1–1.5 mM. After scratching, cells were washed once with PBS to remove debris and then incubated for 24 h with a serum-free medium (SFM). Images of cells were acquired at ×40 magnification with an Olympus IX50 microscope system. The migration distance from the initial edge of the gap to the migration front was measured and analyzed using ImageJ software.

### Live-cell single-cell tracking

Cells overexpressing GFP or GFP-fused Nup88 were seeded with 1–2 × 10^5^ cells on glass-bottom 35 mM dishes (Matsunami Glass, Osaka, Japan) coated with collagen type I (IFP, Yamagata, Japan) and incubated at 37 °C with a standard culture medium in a humidified atmosphere containing 5% CO_2_ until the cells had attached to the bottom. The medium was changed immediately prior to setting the dishes on the stage-top CO_2_ incubator of the microscope. Fluorescence signals from the cells were captured using a Nikon Eclipse TE2000-U inverted microscope system (Tokyo, Japan) at 10-min intervals over a 24-h period. The migration distance of each cell was analyzed using ImageJ software.

### Invasion assay

Invasion assays were performed using a BD Matrigel^**®**^ Invasion Chamber 24-well plate (BD Biosciences, San Jose, CA, USA). T-REx HeLa stable cell lines pre-cultured in medium containing 1 µg/ml doxycycline (DOX) for 24 h were seeded at a density of ~ 1 × 10^5^ cells/well in the upper chamber of the transwell with SFM containing 1 µg/ml DOX. The lower chamber was filled with a standard culture medium containing 1 µg/ml DOX. Invading cells were stained with Diff-Quik reagents (Sysmex, Kobe, Japan) and then counted. An MMP-12 inhibitor, MMP408, was purchased from Merck KGaA (Darmstadt, Germany).

### DNA microarray analysis

Total RNA prepared using an RNeasy Mini Kit (Qiagen, Hilden, Germany) was reverse-transcribed, and the resultant cDNA was hybridized with SurePrint G3 Human Gene Expression 8×60K v2 microarray (Agilent Technologies, Santa Clara, CA, USA) at 65 °C for 17 h with gentle rotation. Gene expression profiling was performed by Medical & Biological Laboratories (Nagoya, Japan).

### Quantitative real-time PCR

Total RNA and cDNA were prepared as outlined earlier. Quantitative real-time polymerase chain reaction (PCR) was performed using SYBR Premix ExTaq II and a Thermal Cycler Dice Real-Time System (Takara Bio Inc., Shiga, Japan). Expression of genes of interest was assessed using the ΔΔ*C*_*t*_ method with 18S rRNA as the internal control. Oligonucleotide primers employed in this assay were as follows: qMMP-12 Fw: 5′-AGTTTTGATGCTGTCACTACCG-3′; qMMP-12 Rv: 5′-CACTGGTCTTTGGTCTCTCAGAA-3′; RN18S1-f: 5′-GCAATTATTCCCCATGAACG-3′; RN18S1-r: 5′-GGGACTTAATCAACGCAAGC-3′.

### Statistical analysis

Differences between mean values for the scratch wound healing assay and transwell invasion assay were evaluated by Student’s *t* test or one-way analysis of variance (ANOVA) with the Tukey–Kramer multiple comparison test. Differences between mean values for live-cell single-cell tracking were evaluated by the Mann–Whitney *U* test after the Shapiro–Wilk test. Differences were significant at *p* < 0.05.

## Results

### Overexpression of Nup88 promotes migration of HeLa cells

**Fig. 1:**
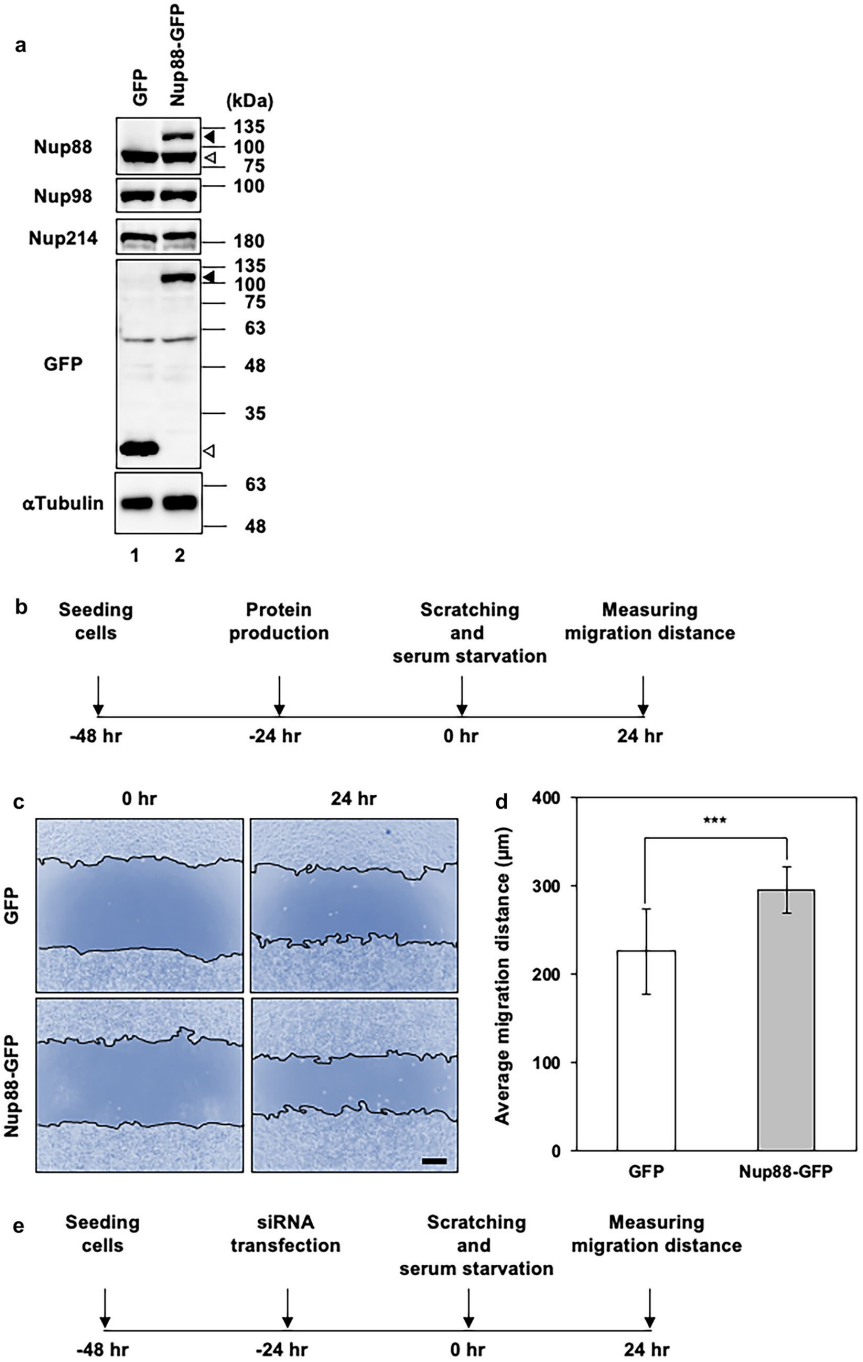

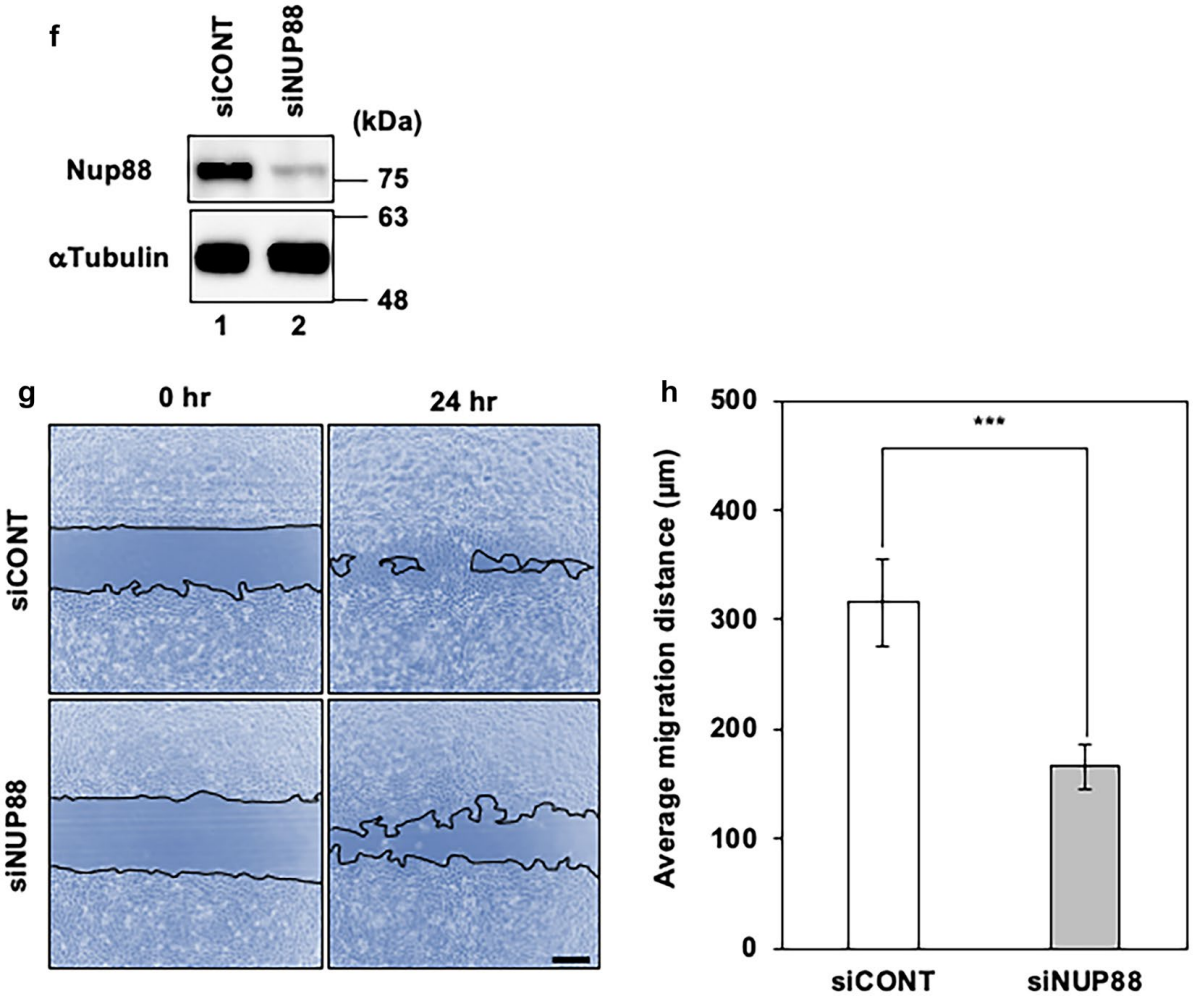
Increased or decreased expression of Nup88 in HeLa cells affects cell migration. **a** Expression of endogenous Nup88, Nup98, and Nup214 in HeLa cells that stably overexpress GFP (lane 1) or Nup88-GFP (lane 2) was analyzed by immunoblotting. Unfilled, lightly shaded, and darker shaded arrowheads indicate GFP, endogenous Nup88, and Nup88-GFP, respectively. α-Tubulin was detected as a loading control. **b** Schematic outline of the scratch wound healing assay for Nup88-overexpressing HeLa cells. A confluent cell layer formed by HeLa cells overexpressing either GFP or Nup88-GFP was scratched to create a straight gap. After scratching, cells were further incubated with SFM for 24 h. **c** Cell migration from the initial gap (0 h) to the migration fronts (24 h) after scratching (scale bar 250 µm). A gap initially created in Nup88-overexpressing cells was completely closed within 24 h. **d** The average migration distance of cells 24 h after scratching was calculated. The average migration distance of GFP- and Nup88-GFP-overexpressing cells was 227 and 297 µm, respectively. Error bars indicate mean ± SD (*n* = 12); ***p < 0.001. **e** Schematic outline of the scratch wound healing assay for Nup88-knockdown HeLa cells. Parental HeLa cells transfected with siRNA were incubated with a serum-containing medium to form a confluent cell layer for 24 h, followed by incubation with SFM for 24 h until just before scratching. After scratching, cells were further incubated with SFM for 24 h. **f** Expression of Nup88 in HeLa cells transfected with control (siCONT) or *NUP88*-targeted (siNUP88) siRNA was analyzed by immunoblotting. α-Tubulin was detected as a loading control. **g** Cell migration from the initial gap (0 h) to the migration fronts (24 h) after scratching (scale bar 250 µm). **h** The average migration distance of cells 24 h after scratching was calculated. The average migration distance of siCONT- and siNUP88-transfected cells was 316 and 166 µm, respectively. Error bars indicate mean ± SD (*n* = 4); ***p < 0.001

Elevated expression of Nup88 has been reported for various types of malignant tumors (Agudo et al. 2004; Zhao et al. 2012; Gould et al. 2002; Emterling et al. 2003). However, whether or not Nup88 overexpression contributes to a malignant phenotype has yet to be determined. Here we examined the effect of the overexpression of Nup88 on both migration and invasion using two previously established HeLa stable cell lines (Makise et al. 2018). First, we confirmed that these cell lines can overexpress GFP or GFP-fused Nup88 (Nup88-GFP) without significantly affecting the expression of either endogenous Nup88 or endogenous binding partners such as Nup98 and Nup214 (Fig. 1a). Next, we evaluated cell migration using a scratch wound healing assay. In this assay, a confluent cell layer that formed on the bottom of a collagen-coated culture plate was scratched to create a straight gap, and then gap closure was monitored (Fig. 1b). We found that the overexpression of Nup88-GFP in HeLa cells slightly, but nonetheless significantly, promoted migration as compared to control HeLa cells overexpressing GFP (Fig. 1c, d). Live-cell single-cell tracking confirmed the enhanced migration of Nup88-overexpressing cells (Supplementary Fig. 1a and Supplementary Videos 1 and 2). To further clarify the involvement of Nup88 in migration, we performed a scratch wound healing assay using Nup88-knockdown HeLa cells (Fig. 1e, f). The knockdown of Nup88 was found to suppress cell migration (Fig. 1g, h). Moreover, comparable Nup88-dependent migration was observed in another cervical cancer cell line, CaSki cells (Supplementary Figs. 1b, 2, and 3, and Supplementary Videos 3 and 4). Taken together, these data indicated that Nup88 stimulates the migration of cervical cancer cells.

### Overexpression of Nup88 promotes invasion of HeLa cells

**Fig. 2:**
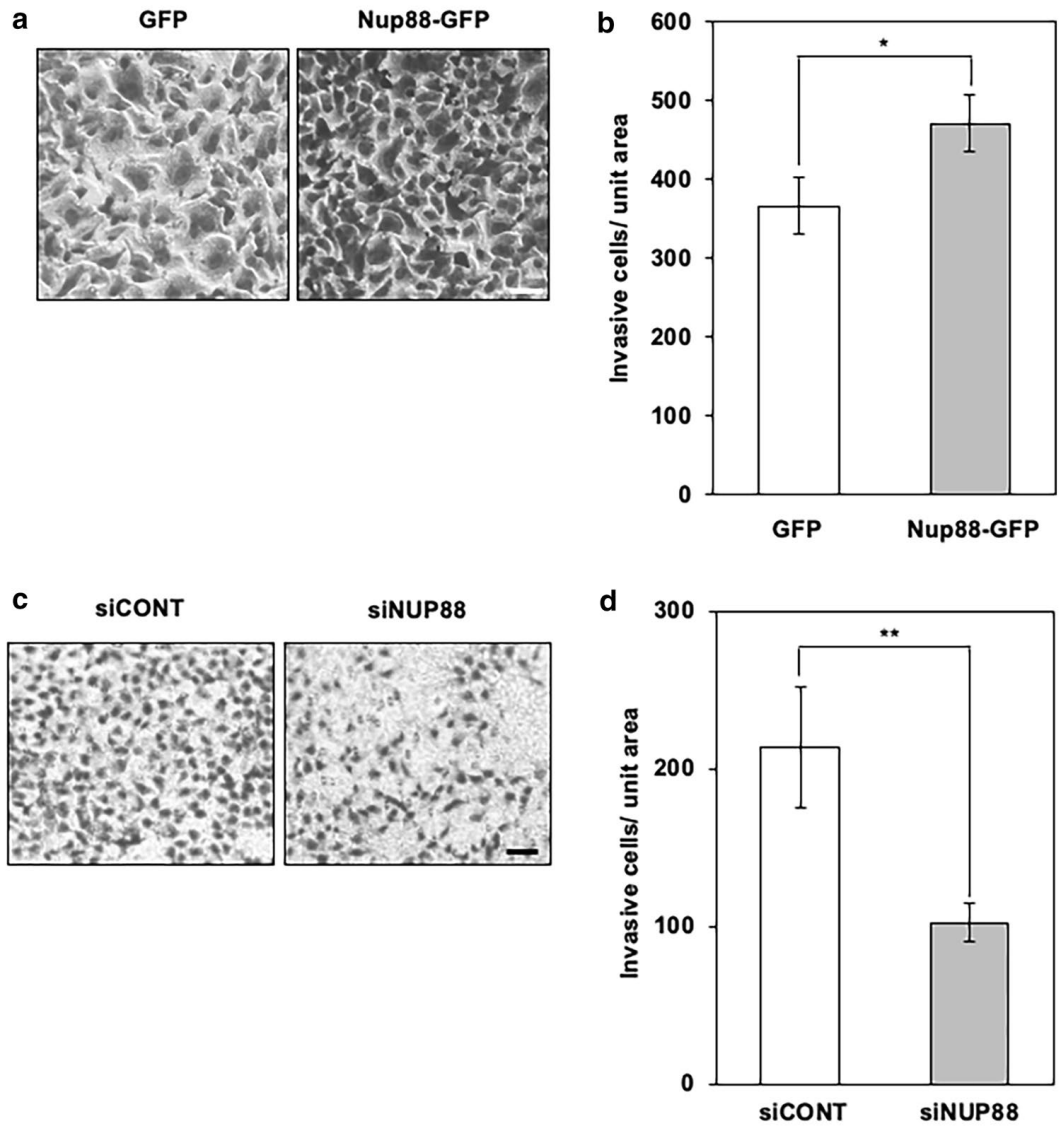
Increased or decreased expression of Nup88 in HeLa cells affects cell invasion. **a** HeLa cells overexpressing either GFP or Nup88-GFP were incubated on an extracellular matrix-coated chamber for 72 h. Invasive cells that migrated through the matrix were subjected to Diff-Quik staining (scale bar 50 nm). **b** The number of invasive cells per unit area was counted. The average number of invasive cells per unit area for cells overexpressing GFP and Nup88-GFP was 366 and 471, respectively. Error bars indicate mean ± SD (*n* = 3); *p < 0.05. **c** HeLa cells transfected with control (siCONT) or *NUP88*-targeted (siNUP88) siRNA were incubated on an extracellular matrix-coated chamber for 72 h. Invasive cells that migrated through the matrix were subjected to Diff-Quik staining (scale bar 50 nm). **d** The number of invasive cells per unit area was counted. The average number of invasive cells per unit area for siCONT- and siNUP88-transfected cells was 214 and 102, respectively. Error bars indicate mean ± SD (*n* = 3); **p < 0.01

Next, to examine the effect of the overexpression of Nup88 on invasion, we performed a Matrigel invasion assay. In this assay, cells were stained and counted after migrating through the Matrigel extracellular matrix. As shown in Fig. 2a, when Nup88-GFP was overexpressed, significantly more cells passed through the matrix compared to cells overexpressing GFP. The subsequent analysis also showed that cells overexpressing Nup88-GFP were 1.29-fold more invasive than those overexpressing GFP (Fig. 2b). In addition, knockdown of Nup88 by siRNA in HeLa cells reduced the number of invasive cells by about 50% as compared to control cells (Fig. 2c, d). In addition to HeLa cells, Nup88-dependent invasion was also observed for CaSki cells (Supplementary Fig. 4). These data indicated that Nup88 can stimulate the invasion of cervical cancer cells.

### NF-κB is not involved in the malignant phenotype caused by Nup88 overexpression

**Fig. 3:**
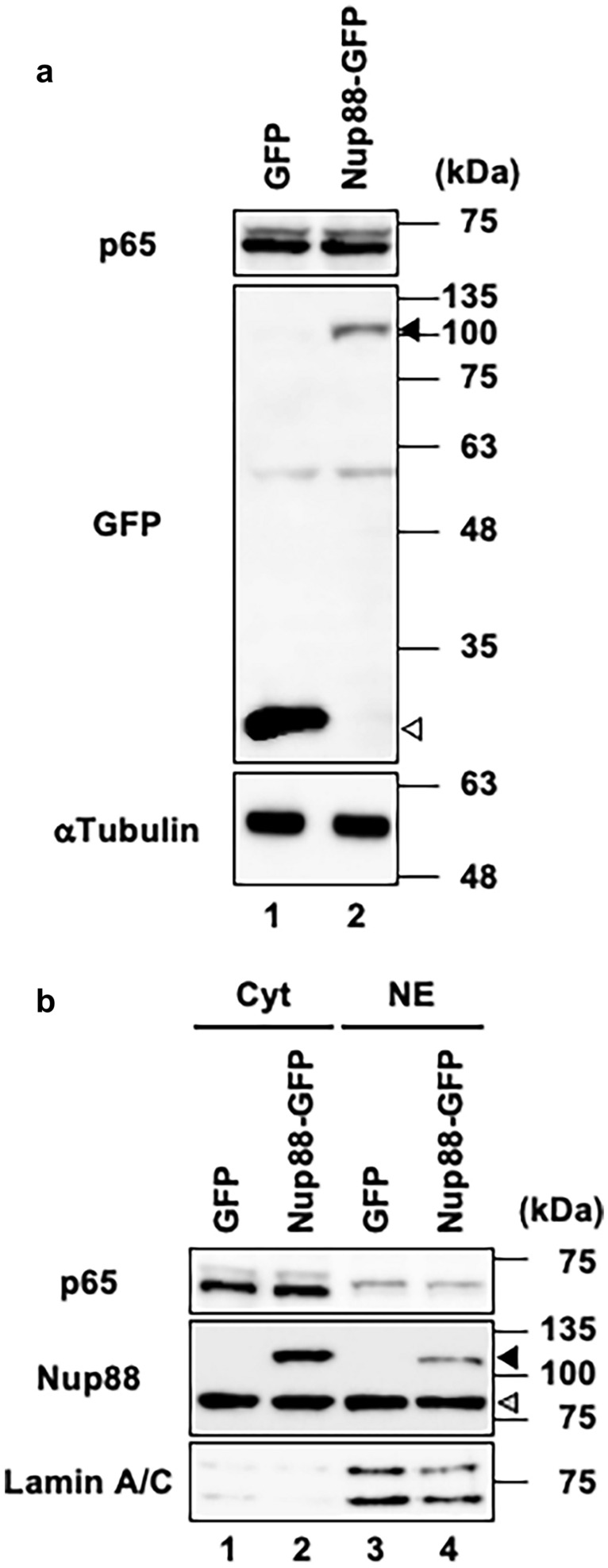
Expression and localization of an NF-κB subunit in HeLa cells overexpressing Nup88. **a** Expression of the p65 subunit of NF-κB in cell lysates prepared from HeLa cells overexpressing GFP or Nup88-GFP was analyzed by immunoblotting. Unfilled and darker shaded arrowheads indicate GFP and Nup88-GFP, respectively. α-Tubulin was detected as a loading control. **b** Localization of the p65 subunit in HeLa cells overexpressing GFP or Nup88-GFP. The p65 subunit was detected in the cytoplasmic fraction (Cyt) and nuclear extract (NE) by immunoblotting. Unfilled and darker shaded arrowheads indicate GFP and Nup88-GFP, respectively. Lamin A/C is an indicator that nuclear/cytoplasmic fractionation was successfully performed

NF-κB is a transcriptional factor that is known to regulate a wide variety of biological processes including migration and invasion (Dolcet et al. 2005). Translocation of NF-κB from the cytosol into the nucleus is thought to be regulated by Nup88 at the level of nucleocytoplasmic transport (Takahashi et al. 2008). We therefore reasoned that the malignant phenotype induced by overexpression of Nup88 could be due to an accumulation of NF-κB in the nucleus. To examine this possibility, we tracked the p65 subunit of NF-κB in Nup88-overexpressing cells. However, contrary to expectation, the overexpression of Nup88 neither affected the expression level of p65 nor promoted its translocation into the nucleus (Fig. 3). Similar results were obtained for CaSki cells (Supplementary Fig. 5). Thus, NF-κB is not involved in the malignant phenotype induced by Nup88 overexpression.

### Overexpression of Nup88 does not stimulate EMT and vice versa

**Fig. 4:**
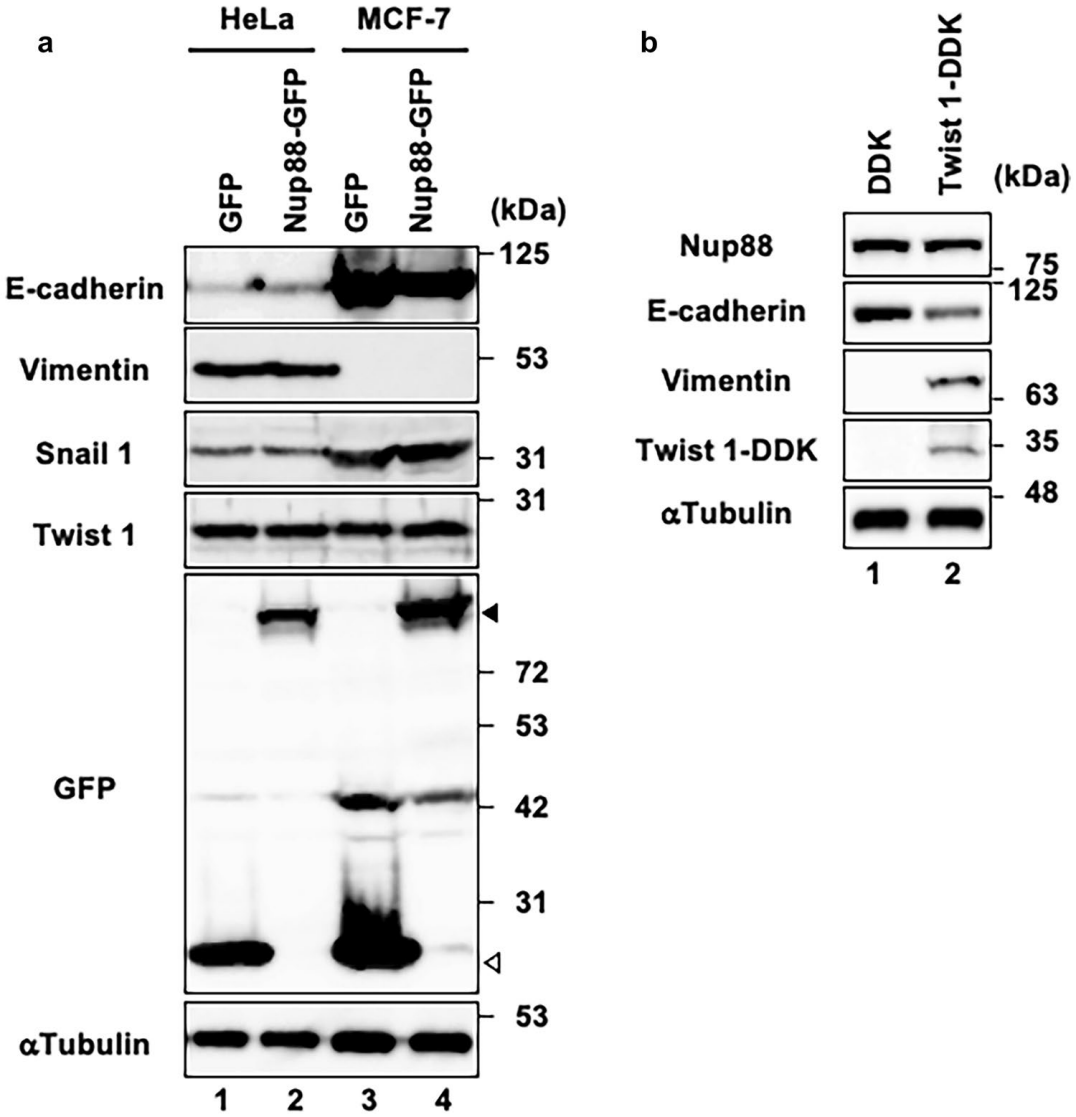
Overexpression of Nup88 does not stimulate EMT and vice versa. **a** Expression of EMT-associated proteins in both HeLa cells (lanes 1 and 2) and MCF-7 cells (lanes 3 and 4) overexpressing GFP (lanes 1 and 3) or Nup88-GFP (lanes 2 and 4) was assessed by immunoblotting. Filled and unfilled arrowheads indicate Nup88-GFP and GFP, respectively. **b** Expression of endogenous Nup88 in both EMT-uninduced (DDK, lane 1) and EMT-induced (Twist-DDK, lane 2) MCF-7 cells was assessed by immunoblotting. α-Tubulin was detected as a loading control

EMT is a critical process for the malignant transformation of tumor cells of epithelial origin (Heerboth et al. 2015). We surmised that the overexpression of Nup88 may induce a malignant phenotype by stimulating EMT progression. EMT is associated with alterations in the expression pattern in a range of different proteins. One such example is E-cadherin, a cell surface glycoprotein that is critical for maintaining cell–cell contact. During EMT the expression of E-cadherin is downregulated by transcriptional repressors such as Snail, Twist, and Zeb (Wheelock et al. 2008). By contrast, the expression of vimentin, which is a type III intermediate filament protein, is upregulated during EMT. To ascertain whether overexpression of Nup88 triggers EMT, we monitored the expression levels of EMT-related proteins in Nup88-overexpressing HeLa cells. As shown in Fig. 4a, the overexpression of Nup88 in HeLa cells did not alter the expression of E-cadherin, vimentin, Snail1, or Twist1 (Fig. 7a, lanes 1 and 2). The same results were obtained when Nup88 was overexpressed in a breast cancer cell line MCF-7, which is epithelial-like well-differentiated cells (Fig. 4a, lanes 3 and 4). These data indicated that overexpression of Nup88 per se does not induce EMT progression. Conversely, we examined the expression of endogenous Nup88 during EMT progression. In this scenario, Twist1 transcriptional repressor was overexpressed in MCF-7 cells to provoke EMT. By comparison to the control transfectant, overexpression of DDK-tagged Twist1 (DDK-Twist1) in MCF-7 resulted in reduced and increased expression of E-cadherin and vimentin, respectively (Fig. 4b). These observations indicated successful induction of EMT in MCF-7 cells. Intriguingly, however, the expression of endogenous Nup88 remained unaltered under these conditions. Taken together, these data indicated that the overexpression of Nup88 does not stimulate EMT and vice versa*.* Therefore, we concluded that the malignant phenotypes observed in Figs. 1 and 2 were not due to EMT progression.

### Overexpression of Nup88 in HeLa cells induces MMP-12 expression at both the gene and protein levels

**Fig. 5:**
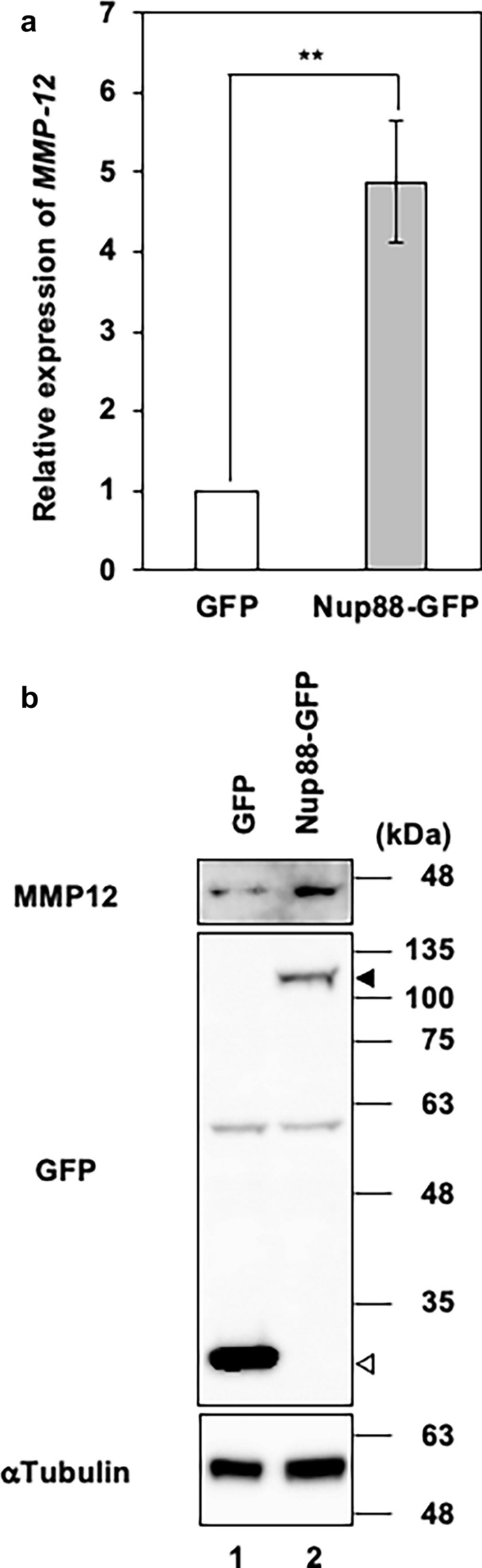
Gene and protein expression of MMP-12 in HeLa cells overexpressing Nup88. **a** Gene expression of MMP-12 in HeLa cells overexpressing either GFP or Nup88-GFP was analyzed by RT real-time PCR. **b** MMP-12 secreted into the culture medium from HeLa cells overexpressing either GFP or Nup88-GFP was analyzed by immunoblotting. Filled and unfilled arrowheads indicate Nup88-GFP and GFP, respectively. α-Tubulin was detected as a loading control

**Fig. 6:**
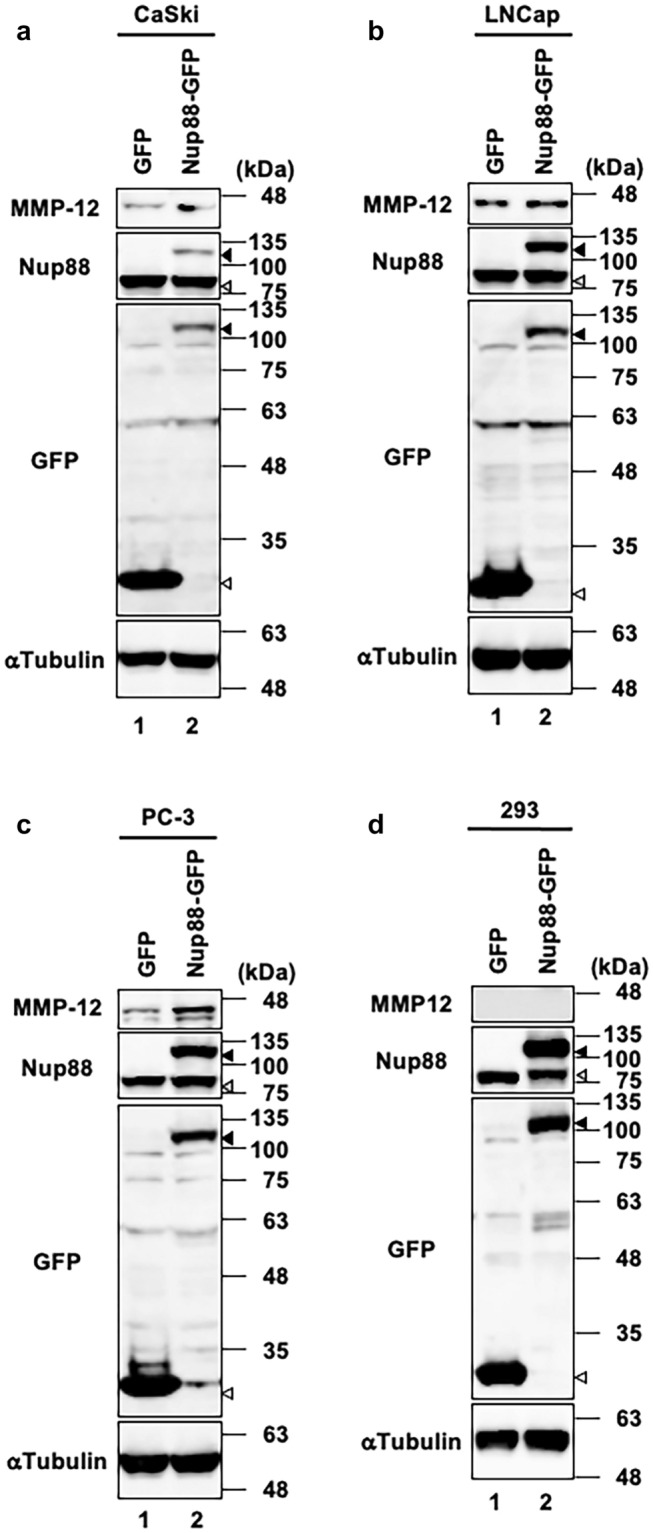
Increased expression of MMP-12 by Nup88 is dependent on cell type. MMP-12 expression in **a** CaSki, **b** LNCap, **c** PC-3, and **d** 293 cells that overexpress GFP or Nup88-GFP either stably (**a** and **d**) or transiently (**b** and **c**) was monitored by immunoblotting. Filled and unfilled arrowheads indicate Nup88-GFP and GFP, respectively. α-Tubulin was detected as a loading control

Given that upregulated MMPs are closely associated with invasion and metastasis of cancer cells, we decided to investigate whether malignant phenotypes induced by Nup88 (Figs. 1, 2) are related to MMPs. At least 23 MMPs are known to exist in human cells, and most of them have been reported to be regulated at the transcriptional level (Egeblad and Werb 2002; Yan and Boyd 2007). We, therefore, quantified the gene expression level of MMPs by DNA microarray analysis in HeLa cells overexpressing either Nup88-GFP or GFP as a negative control. Of the 23 *MMPs* listed in Table 1, 15 and 8 *MMPs* displayed increased and decreased expression, respectively, upon overexpression of Nup88-GFP. We focused on MMP-12 because it showed the most pronounced increase in expression (6.38-fold higher between Nup88-GFP and GFP). This increased level of expression was also confirmed by quantitative real-time PCR analysis (Fig. 5a). We then examined the protein expression of MMP-12. Most MMPs, including MMP-12, are secreted into the extracellular space prior to activation via proteolysis. Thus, we detected MMP-12 present in the culture medium. The observed increase in the protein level of MMP-12 was consistent with the upregulation in gene expression (Fig. 5b). These findings indicated that the protein expression of MMP-12 was increased in a Nup88 overexpression-dependent manner. Interestingly, increased expression of MMP-12 by Nup88 was also observed in CaSki cells and two prostate cancer cell lines (LNCap and PC-3 cells), but not in human embryonic kidney 293 cells (Fig. 6). Moreover, we found that the expression level of exogenous Nup88 was not necessarily proportional to the expression level of MMP-12. For instance, in CaSki cells, exogenous expression of Nup88 was much lower than that of endogenous Nup88 (Fig. 6a), but MMP-12 expression was increased. However, in 293 cells, exogenous Nup88 expression was higher than that of endogenous Nup88, but the expression of MMP-12 was not promoted (Fig. 6d). These data indicated that the response to the overexpression of Nup88 might vary among cell types.

**Table 1:** Gene expression of *MMP*s upon overexpression of Nup88 in HeLa cells

*MMPs*	Fold change (Nup88-GFP vs. GFP)
*MMP-1*	+1.68
*MMP-2*	+1.52
*MMP-3*	+1.49
*MMP-7*	−2.19
*MMP-8*	+1.47
*MMP-9*	−1.11
*MMP-10*	+1.48
*MMP-11*	−2.86
*MMP-12*	+6.38
*MMP-13*	+1.47
*MMP-15*	+1.12
*MMP-14*	−1.58
*MMP-16*	+1.49
*MMP-17*	−1.07
*MMP-19*	+1.43
*MMP-20*	+1.47
*MMP-21*	+1.39
*MMP-23*	−1.18
*MMP-24*	−7.40
*MMP25*	−1.15
*MMP-26*	+1.48
*MMP-27*	+1.49
*MMP-28*	+1.01

Positive and negative values indicate an increase or decrease in gene expression of MMPs for HeLa cells overexpressing Nup88-GFP versus GFP, respectively

### MMP-12 is responsible for invasion caused by the overexpression of Nup88 in HeLa cells

**Fig. 7:**
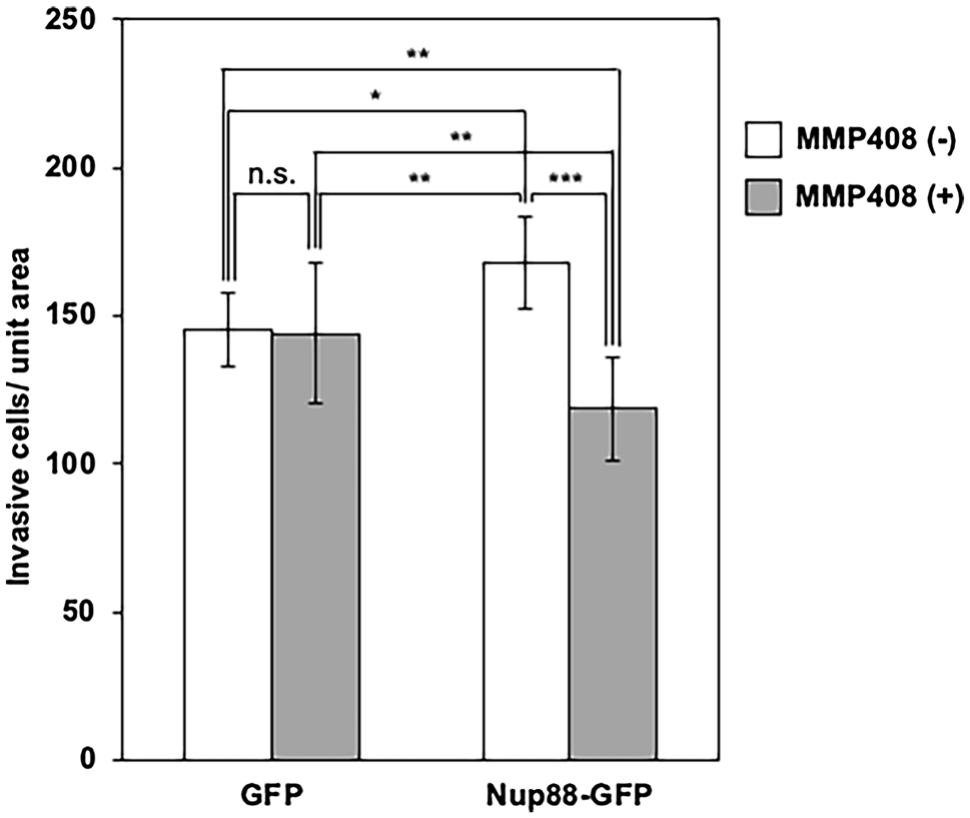
MMP-12 induced by Nup88 stimulates the invasive ability of HeLa cells. HeLa cells overexpressing either GFP or Nup88-GFP were incubated on an extracellular matrix-coated chamber for 48 h in the absence or presence of 10 nM MMP408. The number of invasive cells per unit area was counted. The average number of invasive cells that overexpressed GFP and Nup88-GFP in the absence of MMP408 (unfilled bars) was 145 and 168, respectively, and in the presence of MMP408 (shaded bars) was 144 and 119 cells, respectively. Error bars refer to ± SD (*n* = 3). *n.s.* not significant. *p < 0.05, **p < 0.01, ***p < 0.001

To determine whether MMP-12 is involved in the malignant phenotype caused by Nup88, we assessed the invasive ability of HeLa cells using a Matrigel invasion assay in the absence or presence of MMP408, a selective inhibitor of MMP-12 (Li et al. 2009). In the absence of MMP408, the number of invasive cells overexpressing Nup88 was approximately 1.2-fold higher than that of invasive cells overexpressing GFP (Fig. 7). This finding was broadly consistent with the results shown in Fig. 2. However, in the presence of MMP408, the number of invasive cells overexpressing GFP remained the same, while those overexpressing Nup88 were significantly decreased (Fig. 7). These observations indicated that MMP-12 participates in the Nup88-dependent invasion of HeLa cells.

## Discussion

Several independent lines of evidence indicate that elevated expression of Nup88 is linked with cancer. Nonetheless, there has been a paucity of research focusing on how Nup88 contributes to malignancy, even at the cellular level. The aim of this study was to explore the direct association between Nup88 overexpression and malignant phenotypes in cervical cancer cells.

Our findings suggest that neither NF-κB nor EMT are involved in the promotion of the malignant phenotype caused by Nup88 overexpression in HeLa cells. Activation of NF-κB is known to be associated with the biological process leading to cancer progression. Indeed, nuclear accumulation of activated NF-κB is thought to be regulated by Nup88 at the level of nucleocytoplasmic transport (Takahashi et al. 2008). However, Nup88-overexpressing cells did not accumulate NF-κB in the nucleus under the experimental conditions used in this study (Fig. 3). This result is probably dependent upon whether or not the cells are treated with tumor necrosis factor (TNF). Although TNF is known to be a stimulus for the NF-κB signaling pathway that promotes the nuclear translocation of activated NF-κB, we performed the experiments without TNF treatment (except for serum-derived TNF). Treatment of cells with TNF would probably make it easy to detect the Nup88-dependent nuclear accumulation of NF-κB. Importantly, however, Nup88-dependent migration and invasion were observed without the addition of TNF (Figs. 1, 2). Moreover, EMT-related proteins, such as Twist1, Snail1, and vimentin, whose genes are targets for NF-κB (Julien et al. 2007; Pham et al. 2007; Zheng et al. 2005), showed unchanged levels of expression (Fig. 4a). Therefore, additional activation of NF-κB by Nup88 overexpression did not appear to be required for Nup88-dependent migration and invasion.

It is thought that increased expression of MMPs can act both positively and negatively on tumor progression depending upon the type of cancer cell involved. In clinically isolated cervical carcinomas, some MMPs, including MMP-12, exhibited increased levels of expression. However, the role of these MMPs in terms of cancer progression has yet to be fully understood (Vazquez-Ortiz et al. 2005). In this study, we found that the overexpression of Nup88 or its knockdown increased and decreased, respectively, the expression of MMP-12 in HeLa cells (Fig. 5; Supplementary Fig. 6). Furthermore, we showed that Nup88-dependent invasion activity was suppressed by treatment with MMP408, a selective inhibitor of MMP-12. These findings support the idea that MMP-12 contributes to the malignancy of cervical carcinomas. Interestingly, Nup88-overexpressing cells treated with MMP408 displayed reduced levels of invasion in comparison with GFP-overexpressing cells (Fig. 7). We believe that reduced expression of some MMPs caused by Nup88 overexpression is likely responsible for the lower level of invasion. Overexpression of Nup88 in HeLa cells not only markedly increased the expression of *MMP-12* but also greatly decreased the expression of *MMP-7*, *MMP-11*, and *MMP-24* (Table 1). The inhibition of MMP-12 enzymatic activity in these cells may augment the suppressive effect of the decreased expression of MMPs on their overall invasive activity. Hence, the invasive activity of Nup88-overexpressing cells is reduced compared to GFP-overexpressing cells.

Although the mechanism by which Nup88 increases MMP-12 expression in cervical cancer cells remains to be elucidated, we initially reasoned that increased MMP-12 expression caused by Nup88 overexpression might be mediated through the pathway involved in human papillomavirus (HPV)-derived E6/E7 oncogenes. It has been reported that HPV infection is associated with increased expression of MMP-12 (Vazquez-Ortiz et al. 2005). Both HeLa and CaSki cells have integrated HPV-derived E6/E7 oncogenes in their genome. However, we found that prostate cancer cell lines without integration of E6/E7 oncogenes also showed a Nup88-dependent increase in MMP-12 expression (Fig. 6). Furthermore, although most MMPs, including MMP-12, share common *cis*-elements in their promoters that seem to respond to the same stimuli (Yan and Boyd 2007), only MMP-12 showed a marked increase in expression resulting from Nup88 overexpression. Therefore, we assume that there is a unique pathway for Nup88 to induce MMP-12 expression.

It has been reported that in *Drosophila* and human cells, some Nups detach from NPCs and bind to the genome to regulate the transcription of specific genes (Kohler and Hurt 2010). Nup88 was reported to bind silent foci in *Drosophila* as well and has been identified as a chromatin-associated protein in human cells (Capelson et al. 2010; Shiio et al. 2003). The physiological significance has yet to be determined, but if Nup88 is capable of binding to chromatin and modulating gene expression in human cells, the *MMP-12* gene would be a good target for analyzing the function of Nup88 in gene regulation.

In summary, we conclude that overexpression of Nup88 can stimulate migration and invasion activity of cervical cancer cells, contributing to the formation of malignant phenotypes. While the mechanism by which Nup88 promotes migration requires further investigation, we have shown that Nup88 promotes invasion via MMP-12 expression.

## Supplementary Information

Below is the link to the electronic supplementary material.

Supplementary file1 (DOCX 18 kb)

Supplementary file2 (TIFF 2028 kb)

Supplementary file3 (TIFF 2028 kb)

Supplementary file4 (TIFF 2028 kb)

Supplementary file5 (TIFF 2028 kb)

Supplementary file6 (TIFF 2028 kb)

Supplementary file7 (TIFF 2028 kb)

Supplementary file8 (AVI 51 kb)

Supplementary file9 (AVI 54 kb)

Supplementary file10 (AVI 50 kb)

Supplementary file11 (AVI 83 kb)

## Data Availability

The raw data and materials generated in this study are available from the corresponding author upon reasonable request.
