# Supplementary material for: Overexpression of the nucleoporin Nup88 stimulates migration and invasion of HeLa cells
Source: Histochem Cell Biol. 2021 Jul 31;156(5):409–21. doi: 10.1007/s00418-021-02020-w (PMC8604841; doi:10.1007/s00418-021-02020-w)
Supplement: Supplementary file 1 — Supplementary file1 (DOCX 18 kb) [file 418_2021_2020_MOESM1_ESM.docx]

**Supplementary Videos and Figures legends**

**Supplementary Video 1.**

**Live-cell single-cell tracking of HeLa cells overexpressing GFP**

Live-cell single-cell tracking was performed as described in Materials and Methods. Hourly time-lapse images (25 images) were edited into an 8.3-second video. Supplementary Fig. 1a was generated based on the data.

**Supplementary Video 2.**

**Live-cell single-cell tracking of HeLa cells overexpressing Nup88-GFP**

Live-cell single-cell tracking was performed as described in Materials and Methods. Hourly time-lapse images (25 images) were edited into an 8.3-second video. Supplementary Fig. 1a was generated based on the data.

**Supplementary Video 3.**

**Live-cell single-cell tracking of CaSki cells overexpressing GFP**

Live-cell single-cell tracking was performed as described in Materials and Methods. Hourly time-lapse images (25 images) were edited into an 8.3-second video. Supplementary Fig. 1b was generated based on the data.

**Supplementary Video 4. 
Live-cell single-cell tracking of HeLa cells overexpressing Nup88-GFP**

Live-cell single-cell tracking was performed as described in Materials and Methods. Hourly time-lapse images (25 images) were edited into an 8.3-second video. Supplementary Fig. 1b was generated based on the data.

**Supplementary Fig. 1.**

**Live-cell single-cell tracking of Nup88-overexpressing cells**

(a) Migration velocity of HeLa cells overexpressing GFP or Nup88-GFP. The average migration velocity of cells overexpressing GFP (n=78) and Nup88 (n=94) was 1.64 and 2.10 µm/hr, respectively. (b) Migration velocity of CaSki cells overexpressing GFP or Nup88-GFP. The average migration velocity of cells overexpressing GFP (n=46) and Nup88 (n=35) was 10.2 and 15.0 µm/hr, respectively. Images for these cell lines were taken at 10-min intervals over a 24 hr duration at 37˚C in a serum-containing medium (Supplementary Videos 1 and 2 for HeLa cells, and 3 and 4 for CaSki cells). The images were analyzed using Image J software. Error bars and asterisk (*) mean ± SD and *p*<0.05, respectively.

**Supplementary Fig. 2.**

**Overexpression of Nup88 in CaSki cells promotes cell migration**

(a) Schematic outline of the scratch wound healing assay. A confluent cell layer formed by CaSki cells overexpressing either GFP or Nup88-GFP in a collagen-coated culture plate was scratched to create a wound. After scratching, cells were further incubated with SFM for 24 hr. (b) Cell migration from the initial gap (0 hr) to the migration fronts (24 hr) after scratching (scale bar 250 µm). (c) The average migration distance of cells 24 hr after scratching was calculated. The average migration distance of GFP and Nup88-GFP overexpressing cells was 251 and 304 mm, respectively. Error bars and asterisk (*) mean ± SD (n=5) and *p*<0.05, respectively.

**Supplementary Fig. 3.**

**Knockdown of Nup88 in CaSki cells suppresses cell migration**

(a) Schematic outline of the scratch wound healing assay. Parental CaSki cells transfected with siRNA were incubated with a serum-containing medium for 24 hr to form a confluent cell layer in a collagen-coated culture plate, followed by scratching to make a straight gap. After scratching, cells were further incubated with SFM for 24 hr to monitor wound closure. (b) Expression of Nup88 in CaSki cells after transfection with the control (siCONT) or the *NUP88*-targeted (siNUP88) siRNA. αTubulin was detected as a loading control. (c) Cell migration from the initial gap (0 hr) to the migration fronts (24 hr) after scratching (scale bar 250 µm). (d) The average migration distance of cells 24 hr after scratching was calculated. The average migration distance of siCONT- and siNUP88-transfected cells was 281 and 142 µm, respectively. Error bars and asterisk (***) mean ± SD (n=6) and *p*<0.001, respectively.

**Supplementary Fig. 4.**

**Overexpression of Nup88 in CaSki cells promotes cell invasion**

(a) CaSki cells overexpressing either GFP or Nup88-GFP were incubated on an extracellular matrix-coated chamber for 72 hr. Invasive cells that migrated through the matrix were subjected to Diff-Quik staining (scale bar 50 nm). (b) The number of invasive cells per unit area was counted. The average number of invasive cells per unit area for cells overexpressing GFP and Nup88-GFP were 59 and 94, respectively. Error bars and asterisk (*) mean ± SD (n=3) and *p*<0.05, respectively. (c) CaSki cells transfected with control (siCONT) or NUP88-targeted (siNUP88) siRNA were incubated on an extracellular matrix-coated chamber for 72 hr. Invasive cells that migrated through the matrix were subjected to Diff-Quik staining (scale bar 50 nm). (d) The number of invasive cells per unit area was counted. The average number of invasive cells per unit area for siCONT and siNUP88-transfected cells were 148 and 194, respectively. Error bars and asterisk (**) mean ± SD (n=6) and *p*<0.01, respectively.

**Supplementary Fig. 5.**

**Expression and localization of an NF-κB subunit in CaSki cells overexpressing Nup88**

(a) Expression of the p65 subunit of NF-κB in cell lysates prepared from CaSki cells overexpressing GFP or Nup88-GFP was analyzed by immunoblotting. Unfilled and darker shaded arrowheads indicate GFP and Nup88-GFP, respectively. αTubulin was detected as a loading control. (b) Localization of the p65 subunit in CaSki cells overexpressing GFP or Nup88-GFP. The p65 subunit was detected in the cytoplasmic fraction (Cyt) and nuclear extract (NE) by immunoblotting. Unfilled and darker shaded arrowheads indicate GFP and Nup88-GFP, respectively. Lamin A/C is an indicator that nuclear/cytoplasmic fractionation was successfully performed.

**Supplementary Fig. 6.**

**Expression of MMP-12 in Nup88 depleted HeLa cells**

(a) MMP-12 expression in HeLa cells transfected with control (siCONT) and *NUP88*-targeted (siNUP88) siRNA was assessed by immunoblotting. αTubulin was detected as a loading control. (b) Relative expression of MMP-12 in Nup88-depleted HeLa cells. Data were collected from three independent experiments including the result shown in (a). Error bars and asterisk (**) mean ± SD and *p*<0.001, respectively.
