# Supplementary figures and images for: Overexpression of the nucleoporin Nup88 stimulates migration and invasion of HeLa cells
Source: Histochem Cell Biol. 2021 Jul 31;156(5):409–21. doi: 10.1007/s00418-021-02020-w (PMC8604841; doi:10.1007/s00418-021-02020-w)

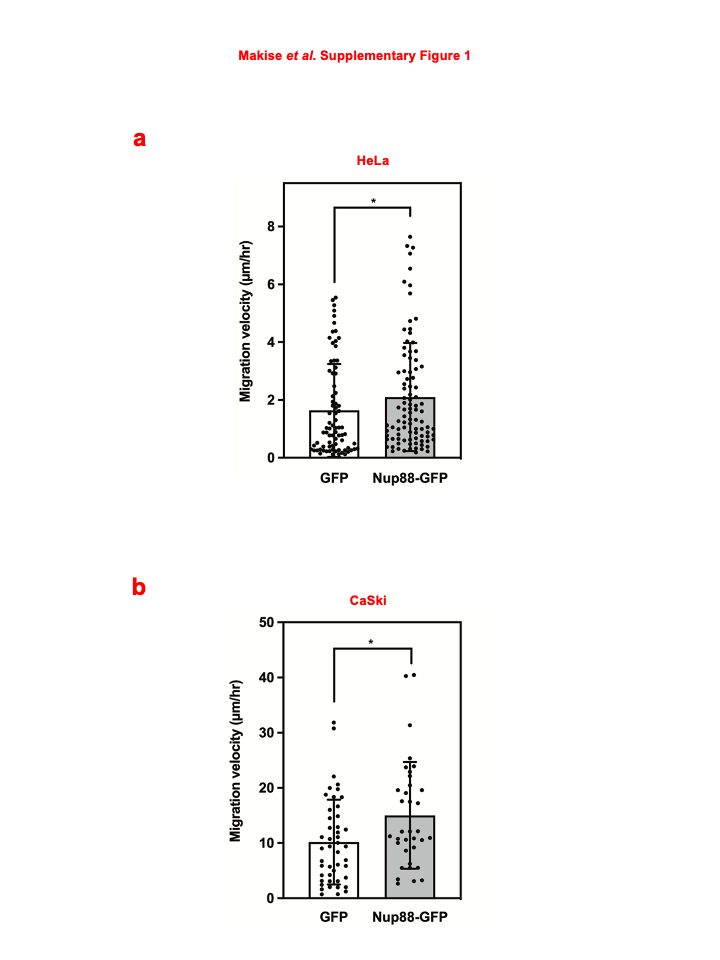

Supplement: Supplementary file 2 — Supplementary file2 (TIFF 2028 kb) [file 418_2021_2020_MOESM2_ESM.tiff]

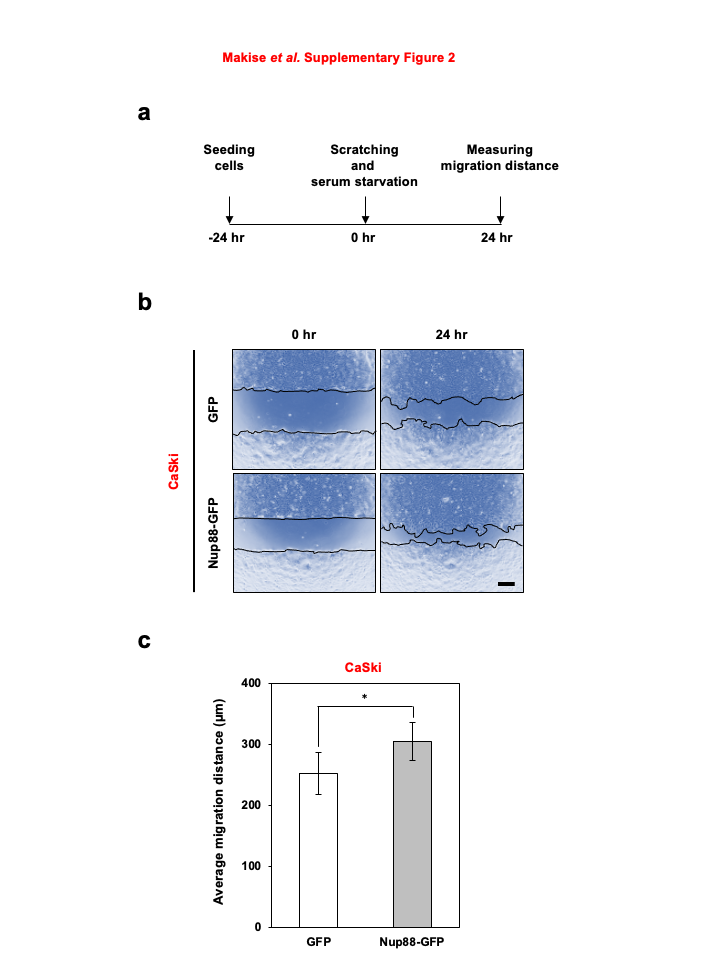

Supplement: Supplementary file 3 — Supplementary file3 (TIFF 2028 kb) [file 418_2021_2020_MOESM3_ESM.tiff]

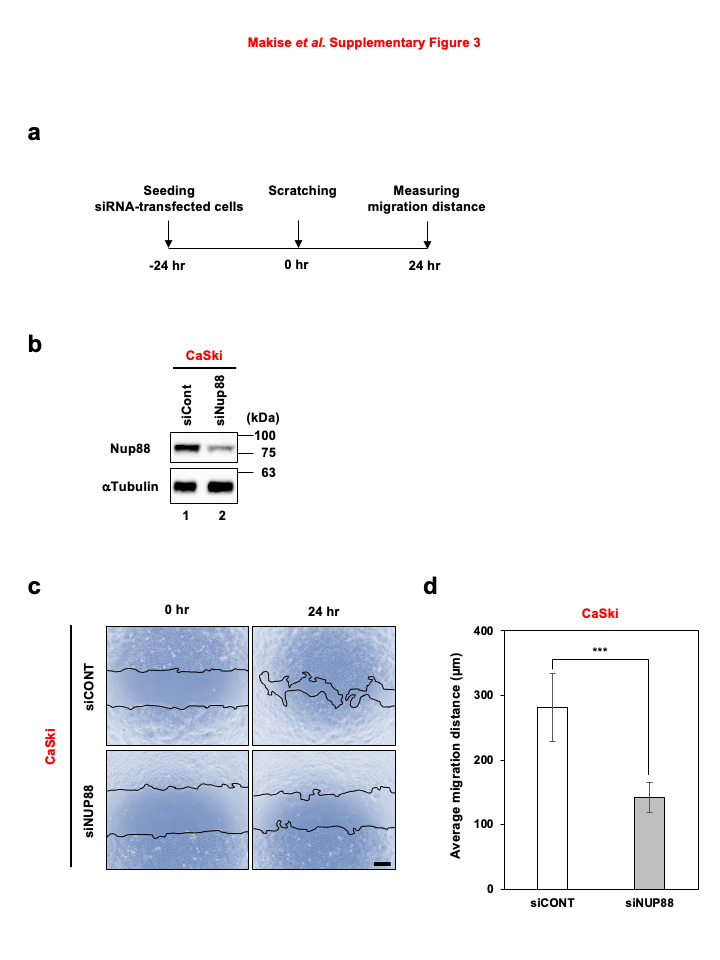

Supplement: Supplementary file 4 — Supplementary file4 (TIFF 2028 kb) [file 418_2021_2020_MOESM4_ESM.tiff]

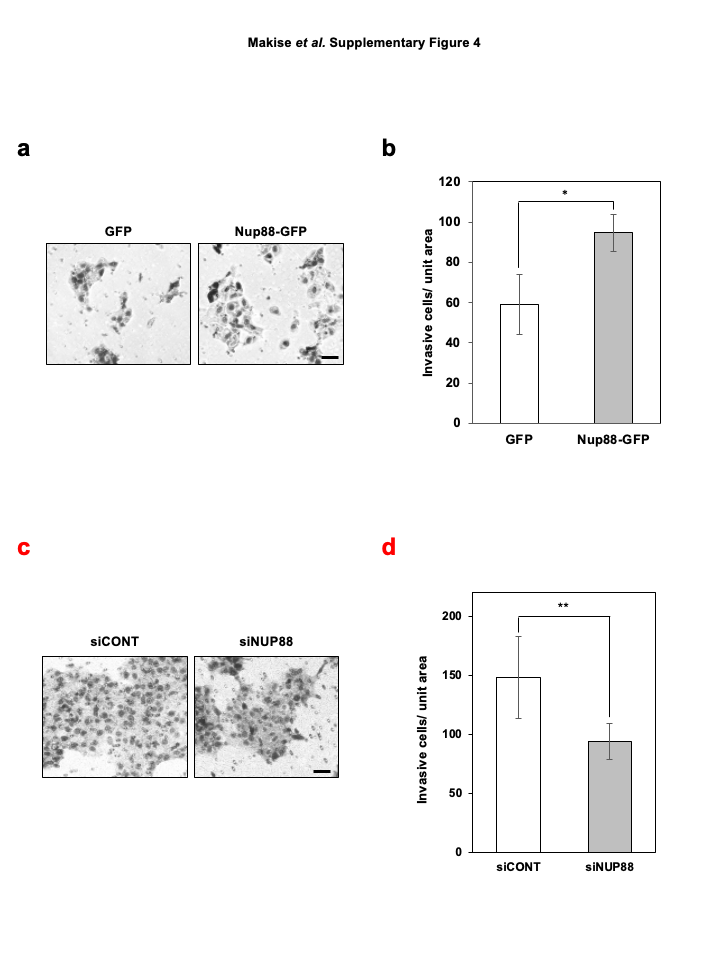

Supplement: Supplementary file 5 — Supplementary file5 (TIFF 2028 kb) [file 418_2021_2020_MOESM5_ESM.tiff]

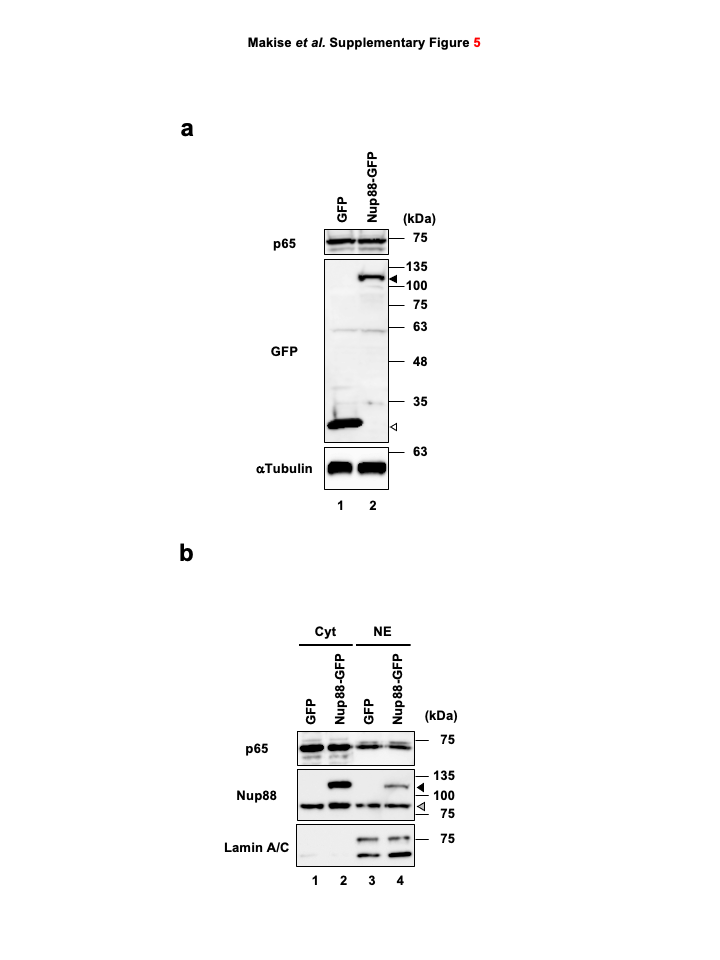

Supplement: Supplementary file 6 — Supplementary file6 (TIFF 2028 kb) [file 418_2021_2020_MOESM6_ESM.tiff]

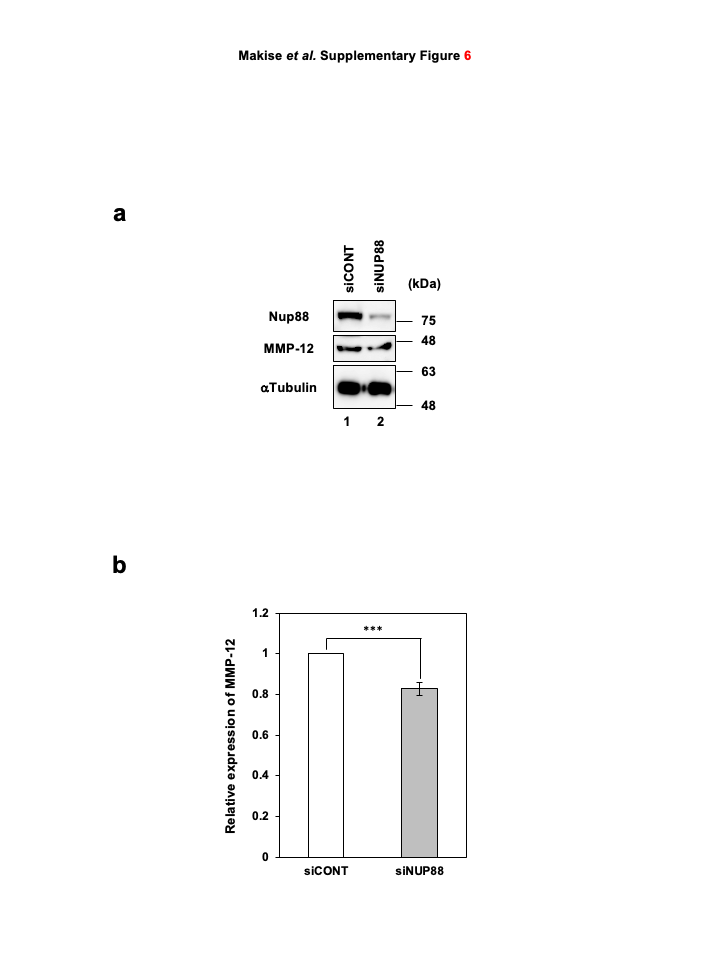

Supplement: Supplementary file 7 — Supplementary file7 (TIFF 2028 kb) [file 418_2021_2020_MOESM7_ESM.tiff]
